# DNA methylation profiling in the thalamus and hippocampus of postnatal malnourished mice, including effects related to long-term potentiation

**DOI:** 10.1186/1471-2202-15-31

**Published:** 2014-02-20

**Authors:** Xiaoling Weng, Daizhan Zhou, Fatao Liu, Hong Zhang, Junyi Ye, Zhou Zhang, Di Zhang, Yinan Wang, Liming Tao, Lan Cao, Mengyuan Kan, Ting Wang, Guoyin Feng, Xiaolan Qin, Jihui Sun, Lin He, Yun Liu

**Affiliations:** 1 Institutes of Biomedical Sciences, Fudan University, Shanghai 200032, PR China; 2 Bio-X Center, Key Laboratory for the Genetics of Developmental and Neuropsychiatric Disorders, Ministry of Education, Shanghai Jiao Tong University, Shanghai 200030, PR China; 3 Institute for Nutritional Sciences, Shanghai Institutes for Biological Sciences, Chinese Academy of Sciences, Shanghai 200031, PR China; 4 Luwan Branch of Ruijin Hospital, Shanghai Jiaotong University School of Medicine, Shanghai 200030, PR China; 5 Key Laboratory of Molecular Medicine, The Ministry of Education, Department of Biochemistry and Molecular Biology, Fudan University Shanghai Medical College, 303 Ming Dao Building, 138 Yi Xue Yuan Road, Shanghai 200032, PR China; 6 Bio-X Institute, Shanghai Jiao Tong University, Small White House, 1954 Hua Shan Road, Shanghai 200030, PR China

**Keywords:** Malnutrition, Thalamus, Hippocampus, Mouse model, Global DNA methylation status, Whole genome methylation sequencing, Long-term potentiation, Psychiatric disorders

## Abstract

**Background:**

DNA methylation has been viewed as the most highly characterized epigenetic mark for genome regulation and development. Postnatal brains appear to exhibit stimulus-induced methylation changes because of factors such as environment, lifestyle, and diet (nutrition). The purpose of this study was to examine how extensively the brain DNA methylome is regulated by nutrition in early life.

**Results:**

By quantifying the total amount of 5-methylcytosine (5mC) in the thalamus and the hippocampus of postnatal malnourished mice and normal mice, we found the two regions showed differences in global DNA methylation status. The methylation level in the thalamus was much higher than that in the hippocampus. Then, we used a next-generation sequencing (NGS)-based method (MSCC) to detect the whole genome methylation of the two regions in malnourished mice and normal mice. Notably, we found that in the thalamus, 500 discriminable variations existed and that approximately 60% were related to neuronal development or psychiatric diseases. Pathway analyses of the corresponding genes highlighted changes for 9 genes related to long-term potentiation (5.3-fold enrichment, P = 0.033).

**Conclusions:**

Our findings may help to indicate the genome-wide DNA methylation status of different brain regions and the effects of malnutrition on brain DNA methylation. The results also indicate that postnatal malnutrition may increase the risk of psychiatric disorders.

## Background

Nutrition represents one of the major important variables that play crucial roles in the maturation and functional development of the central nervous system (CNS) [1]. Malnutrition exerts its effects during the brain growth spurt period, and it results in a variety of brain dysfunctions [2]. Growth deficits due to malnutrition in childhood increase the incidence of infectious diseases and lead to alterations in CNS function, which have been shown to delay psychomotor development [3]. Thus, understanding the pathological effects of malnutrition will provide critical insights into the neurodevelopmental process.

DNA methylation is among the best studied epigenetic modifications and is essential to mammalian development [4]. Specifically, the methylation of cytosine at CpG dinucleotides is an important regulatory modification of the genome [5]. Many epigenetic studies on DNA methylation have revealed that malnutrition during the perinatal period is highly correlated with abnormal neurodevelopment [6].

Studies of epigenetic modifications benefit substantially from improved next-generation sequencing methods, and recent technologies make it possible for accurate and large-scale CpG methylation profiling [7]. Methyl-sensitive cut counting (MSCC) is a NGS method used to profile the whole DNA methylome. The MSCC genome-scale analysis is based on the concept that locations of CCGGs largely reflect the distributions of all CpGs in the mouse genome [8].

In our study, we utilized a mouse model of male mice to avoid sex differences, as sexual dimorphism and sexual differentiation have been thought to underlie the sexual development of the brain and other organs, which could impact lifelong functions [9]. First, we detected the global DNA methylation status in the thalamus and the hippocampus of postnatal malnourished mice and normal mice. Because the two regions showed differences in global DNA methylation status, we employed MSCC to investigate DNA methylation to determine how extensively the two brain DNA methylomes were regulated by nutrition in early life.

## Methods

### Mouse husbandry

All animal care and use procedures were in accordance with the guidelines of the Institutional Animal Care and Use Committee for Nutritional Sciences, Shanghai Institutes for Biological Sciences, Chinese Academy of Sciences. The parental mice in this study were purchased from Shanghai Laboratory Animal Co. Ltd. (SLAC, Shanghai, China) and housed under controlled conditions of 12 h light/12 h dark cycle at 23 ± 2°C and 35 ± 5% humidity. The parental mice had been raised for at least two generations on a control diet to attempt to minimize any trans-generational effects. The male offspring mice were weaned from the mothers at 20 days of age and were divided randomly into two groups (n = 7 in control group, n = 5 in famine group): the control group was given a standard diet (D12450B), and the famine group was fed with half of a low-protein diet (D06022301) [10]. After 6 weeks of artificial feeding, offspring mice were euthanized (the weights of the offspring mice were measured every week from weaning); the entire thalamus and hippocampus were dissected out and immediately stored at -80°C.

### DNA preparation

Genomic DNA was isolated from ≥25 mg samples (thalamus and hippocampus of malnourished mice and normal mice) using the QIAamp DNA Mini Kit following the standard protocol (QIAGEN, Hilden, Germany). To ensure the sample quality, a Thermo NanoDrop 2000 (Thermo, Wilmington, USA) was used to detect 260/280 nm UV absorbance ratios, and concentrations were determined with a Qubit 2.0 fluorometer (Life Technologies, Carlsbad, CA). DNA length was determined by an Agilent 2100 Bioanalyzer (Agilent, Santa Clara, CA) to ensure the integrity. We constructed two MSCC libraries for each of our two brain tissues that contained pooled DNA from the control group and the famine group. Pooled DNA was used to increase the sample concentration and diminish micro-dissection variations.

### Global DNA methylation status detection and calculation

The MethylFlash™ Methylated DNA Quantification Kit (Fluorometric) was used to detect the total amount of 5mC in the thalamus and hippocampus according to the manufacturer’s instructions (Epigentek, Farmingdale, NY). Relative fluorescence units (RFU) at 530_EX_/590_EM_nm were measured using a SpectraMax M5 (Molecular Devices, CA, USA) to calculate the global methylation status.

### MSCC library construction

To detect cytosine methylation, MSCC utilized the restriction enzymes *Hpa*II and *Msp*I, which preferentially cut DNA based on its methylation status. Although both enzymes recognized the same restriction site CCGG, *Hpa*II was a methylation-sensitive enzyme, while *Msp*I was insensitive to methylation sites. Adaptors A and B were also created for MSCC and were synthesized by Sangon Biotech (Shanghai, China). Adaptor A contained a 5′ *Mme*I recognition site and a 5′ CG overhang, while adaptor B had a 3′ NN overhang. Both adapters also contained end sequences required for Illumina library construction. Then, we constructed a *Hpa*II library and an Inverse library for our study. To correct the error during the experiment of the *Hpa*II library and the Inverse library, we combined standard DNA in the two libraries that contained the same known methylated or no methylated sequences. Further details are provided in the Additional file 1: Supplementary Method.

### Library sequencing and mapping

Before sequencing, we used a Perl script to extract 20 bp upstream and 20 bp downstream of every CCGG site from the whole mouse genome (mm9). Based on the UCSC Refseq database, we added annotation to these short sequences and established a CCGG sequence database. The two libraries were sequenced on the Hiseq2000 depth sequencing system (Illumina, San Diego, California). After sequencing, the *Hpa*II library and the Inverse library data from the Hiseq2000 depth sequencing system (bcl document) were transformed into a fastq file using CASAVA software. The fastq file data were mapped to the CCGG sequence database that we constructed using MOM software. We then analyzed the number of reads in the same CCGG position to calculate the degree of methylation of each site. Standard DNA in each library was used to normalize the counts before estimating the methylation level, according to the method in Guo et al. [8].

### Statistical analysis

Student’s *t*-tests were used to determine the differences in mouse weights and thalamus and hippocampus methylation levels between the control group and the famine group. For the MSCC data, we constructed a 200-bp non-overlapping windows profiling of the whole genome. Windows that involved more than 3 CpG sites sequenced were used for the subsequent analysis. For each of the 200-bp windows, we conducted Fisher exact tests to find different methylated regions (DMRs) with statistical significance (P < 0.05) and different levels of methylation changes (∆MSCC, either increase or decrease) >25%. A P value < 0.05 was considered significant in these analyses.

### Pathway classification and enrichment analysis

Pathway analyses were performed using the Database for Annotation, Visualization and Integrated Discovery (DAVID, http://david.abcc.ncifcrf.gov/) [11]. Pathway classification within DAVID used the Kyoto Encyclopedia of Genes and Genomes database (KEGG, http://www.genome.jp/kegg/pathway.html) and PANTHER ( http://www.pantherdb.org/pathway/). Enrichment statistics were adjusted by Benjamini correction.

## Results

### Weight

The weights of the offspring male mice are shown in Table 1. Data are presented as the mean ± standard deviation (SD). As determined using Student’s *t*-test, the famine group weighed less and the weights were significantly different from the control group (p < 0.05). During feeding, the famine group displayed the phenomena of mania and hyperactivity.

**Table 1:** Mouse weights from the beginning of artificial feeding

**Group**	**Weight (g)/week**						
	**0**	**1**	**2**	**3**	**4**	**5**	**6**
Control	8.69 ± 0.32	19.06 ± 0.85	21.57 ± 1.21	23.29 ± 1.08	24.53 ± 1.57	25.51 ± 2.02	26.04 ± 1.87
Famine*	8.69 ± 0.27	8.82 ± 0.77	10.28 ± 0.44	12.46 ± 0.50	13.32 ± 0.68	13.02 ± 0.94	11.45 ± 0.78

*P < 0.05, compared with the control group.

### Total 5mc amount in the thalamus and hippocampus

**Figure 1:**
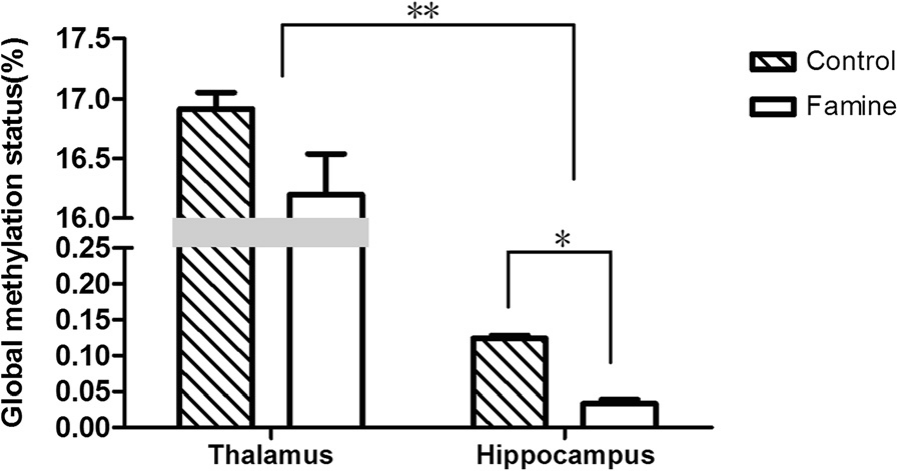
**The global DNA methylation levels in the thalamus and hippocampus of postnatal malnourished mice and normal mice.** Data are the mean ± s.e.m. *P = 0.046 (control group and famine group in the hippocampus, Student’s *t*-test). **P = 6.413e-05 (control group in the thalamus and hippocampus, Student’s *t*-test); 4.394e-04 (famine group in the thalamus and hippocampus, Student’s *t*-test).

The global DNA methylation status in the thalamus and the hippocampus of postnatal malnourished mice and normal mice is shown in Figure 1. The global DNA methylation status in the thalamus was significantly higher than that of the hippocampus. In both the thalamus and the hippocampus, the control group had a higher total 5mc amount than the famine group.

### DNA methylome in the thalamus and hippocampus

**Figure 2:**
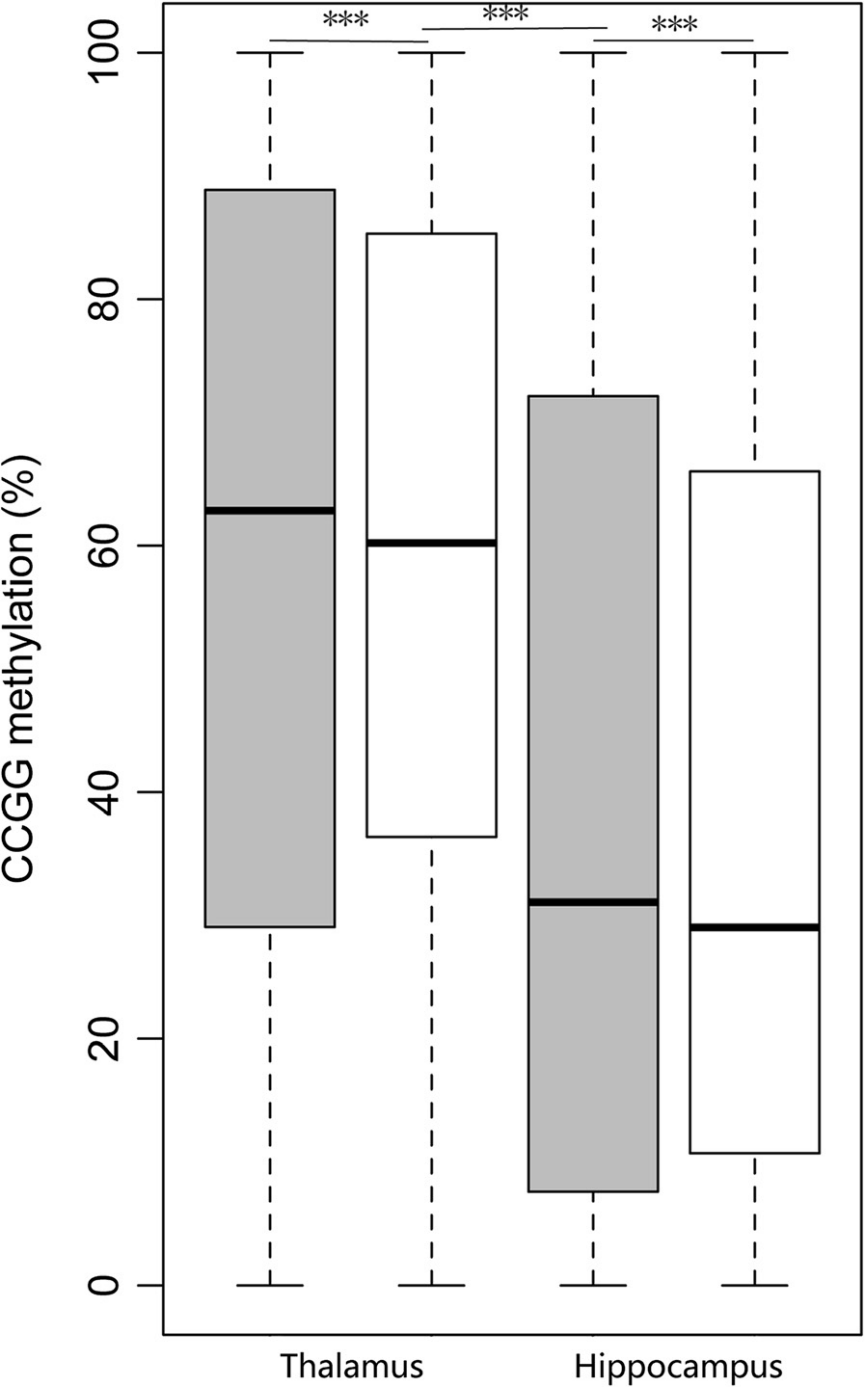
**The overall methylation levels of all CCGG sites in the thalamus and hippocampus (control group in gray and famine group in white).** The total methylation level at CCGG sites of the hippocampus was lower than that of the thalamus. ***P < 0.001 (control group and famine group in the thalamus and hippocampus, control group in the thalamus and hippocampus, famine group in the thalamus and hippocampus, Student’s *t*-test).

The reads obtained from our libraries after MSCC whole genome methylation sequencing are shown in Table 2. After excluding MSCC sites with low sequencing depth, we obtained the DNA methylation profile of the thalamus (Additional file 2: Table S1) and the hippocampus (Additional file 3: Table S2). The overall methylation levels of all CCGG sites in the two tissues are shown in Figure 2. The CCGG methylation level of the hippocampus was also lower than that of the thalamus as the global DNA methylation status. The methylation differences between the famine and control groups or the thalamus and hippocampus were statistically significant (P < 0.001).

**Table 2:** MSCC Illumina sequencing statistics of the thalamus and hippocampus

**Library name**		**Number of reads with appropriate adaptors**	**Number of mapped reads (percentage)**	**Number of CCGG sites seen at least once**	**Average number of reads per CCGG site**
Thalamus	Control-*Hpa*II	39239224	31413443 (80.0)	1034841	30.4
	Control-Inverse	35769649	20466887 (57.2)	1118555	18.3
	Famine-*Hpa*II	57387236	46262199 (80.6)	1076064	43
	Famine-Inverse	46472846	27799576 (59.8)	1175605	23.6
Hippocampus	Control-*Hpa*II	29639848	24301025 (81.2)	1060634	22.9
	Control-Inverse	26585724	15892801 (59.8)	1136208	14.0
	Famine-*Hpa*II	37551216	30429853 (81.0)	1125683	27.0
	Famine-Inverse	42435514	25510407 (60.1)	1191446	21.4

In addition, we also showed the methylation level in internal, shore and external CGIs of the thalamus and the hippocampus (Additional file 1: Figure S1a and S1b). In the two groups of both tissues, the level of methylation in the internal of the CpG islands was lower than that of the external of the CpG islands. Furthermore, the famine group had a lower methylation level than the control group at the external of the CpG islands, whereas it was hypermethylated in the internal of the CpG islands in both brain regions. The whole genome methylation pattern showed that the methylation level near the transcription initiation site (TSS) was lower than any other position (Additional file 1: Figure S2).

We combined the mouse thalamus gene expression data from the NCBI GEO database (GDS1490) with our MSCC thalamus data for the control group. First, we divided the database expression genes into three parts: high-expression genes, moderate-expression genes, and low-expression genes. Then, the MSCC data were integrated to the three parts of the expression genes. The relationship between the methylation level and gene expression is shown in Additional file 1: Figure S3a and S3b. The methylation level of the low-expression genes was higher at TSS but lower at the gene body than that of the high-expression genes.

There were 500 distinct genes in the thalamus (Additional file 4: Table S3) but none in the hippocampus, according to the results of the DMR analysis. Furthermore, all 500 selected genes obtained from the thalamus analysis of the famine group showed hypermethylation, and approximately 60% of the genes were associated with neuronal development and psychiatric diseases. The 20 crucial genes identified as being implicated in psychiatric diseases are shown in Table 3.

**Table 3:** Twenty crucial genes related to psychiatric diseases among 500 selected genes of the thalamus

**Gene name**	**Related psychiatric diseases**
*Slc18a2*	Schizophrenia [12], Alzheimer’s disease [13], Parkinson’s disease [14]
*Pi4k2b*	Bipolar disorder [15]
*Prkacb*	Alzheimer’s disease [16]
*Gabra1*	Schizophrenia [17]
*Bdnf*	Alzheimer’s disease [18], Major depression disorder [19]
*Katnb1*	Bipolar disorder [20]
*Psen1*	Alzheimer’s disease [21]
*Pde4a*	Schizophrenia [22], Bipolar disorder [23]
*Lrfn5*	Autism [24]
*Ppp3ca*	Alzheimer’s disease [25,26]
*Shank3*	Autism spectrum disorder [27,28], Bipolar disorder [29]
*Clu*	Alzheimer’s disease [30,31]
*Notch4*	Bipolar disorder [32], Schizophrenia [33]
*Sult4a1*	Schizophrenia [34]
*Rnf11*	Parkinson’s disease [35]
*Pclo*	Major depression disorder [36], Bipolar disorder [37]
*Tyro3*	Alzheimer’s disease [38]
*Calm2*	Major depression disorder [39]
*Cacna1c*	Schizophrenia [40-42], Bipolar disorder [43-45]
*Plcb1*	Autism spectrum disorder [46], Schizophrenia [47]

### Properties of the hypermethylated genes

**Figure 3:**
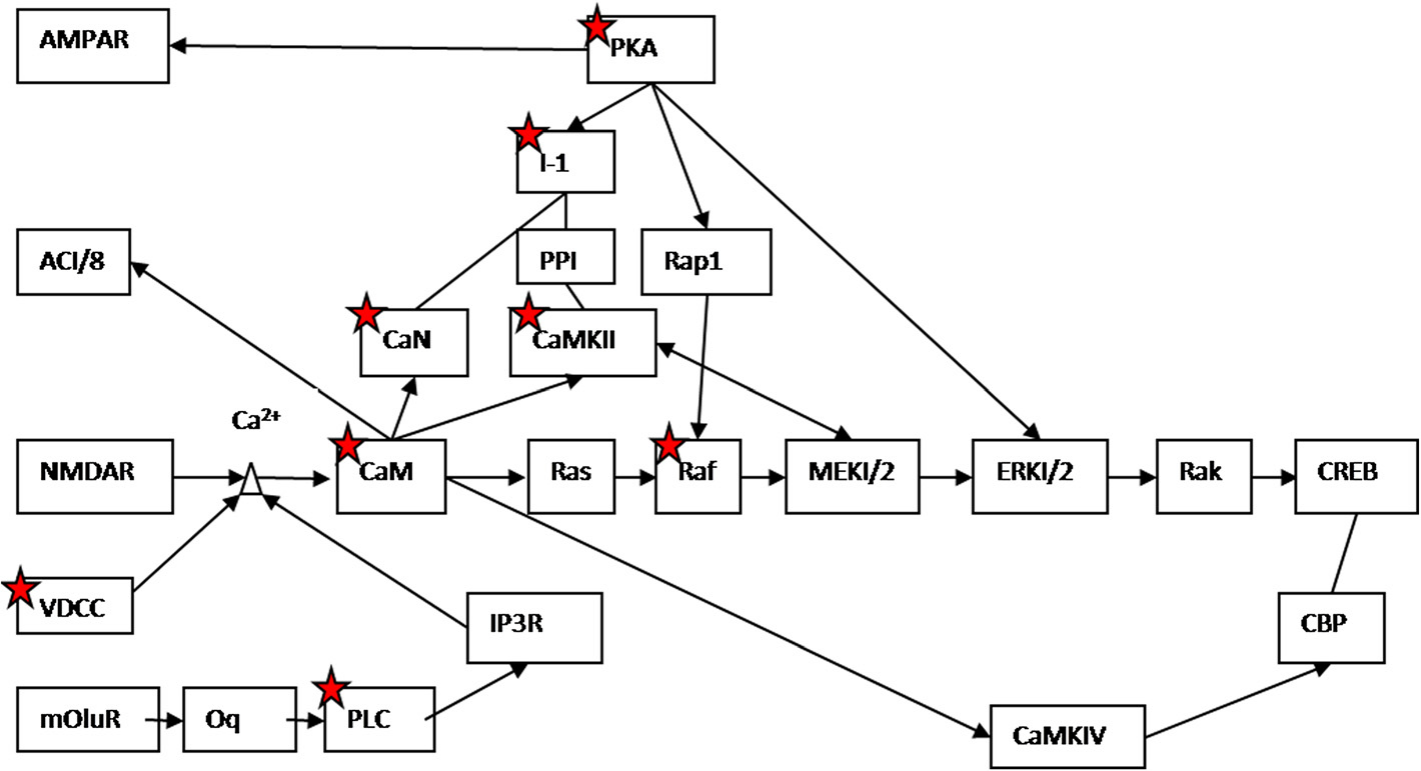
**The 9 relative genes marked with red stars: *Ppp1r1a*** **(I-1), *Ppp3r1*** **(CaN), *Raf1*** **(Raf), *Camk2b*** **(CaMKII), *Ppp3ca*** **(CaN), *Prkacb*** **(PKA), *Cacna1c*** **(VDCC), *Plcb1*** **(PLC), and *Calm2*** **(CaM) in the most significant identified pathway, i.e., long-term potentiation.**

To gain mechanistic insights into the CpG modifications, we constructed our 500 gene set corresponding to biological pathways and networks underlying complex diseases using the online tool DAVID. In total, 20 pathways were matched with our gene sets, as shown in Table 4. The most highly enriched pathway, which remained significant after the adjustment for multiple hypothesis testing using the conservative Benjamini correction, was long-term potentiation (LTP, 5.3-fold enrichment, P = 0.033) with 9 relative genes from our study: *Ppp1r1a*, *Ppp3r1*, *Raf1*, *Camk2b*, *Ppp3ca*, *Prkacb*, *Cacna1c*, *Plcb1*, and *Calm2* (Figure 3).

**Table 4:** Twenty KEGG and PANTHER pathways applied to the 500 hypermethylated genes in the thalamus

**Category**	**Pathway**	**Count**	**Fold**		**Benjamini**
			**Enrichment**	**P value**	**P value**
KEGG_PATHWAY	Long-term potentiation	9	5.270	2.58 × 10-4	0.033
KEGG_PATHWAY	MAPK signaling pathway	16	2.475	0.002	0.108
PANTHER_PATHWAY	Alzheimer disease-amyloid secretase pathway	9	3.872	0.002	0.147
KEGG_PATHWAY	Long-term depression	7	3.985	0.008	0.283
KEGG_PATHWAY	GnRH signaling pathway	8	3.380	0.009	0.250
KEGG_PATHWAY	Calcium signaling pathway	11	2.360	0.017	0.360
KEGG_PATHWAY	Vascular smooth muscle contraction	8	2.732	0.026	0.433
KEGG_PATHWAY	Wnt signaling pathway	9	2.476	0.028	0.405
KEGG_PATHWAY	Melanogenesis	7	2.869	0.034	0.428
PANTHER_PATHWAY	TGF-beta signaling pathway	10	2.080	0.046	0.876
KEGG_PATHWAY	Ubiquitin mediated proteolysis	8	2.411	0.046	0.496
PANTHER_PATHWAY	Endogenous_cannabinoid_signaling	4	4.832	0.047	0.757
PANTHER_PATHWAY	PDGF signaling pathway	11	1.930	0.052	0.698
KEGG_PATHWAY	Glycerophospholipid metabolism	5	3.059	0.078	0.654
PANTHER_PATHWAY	Endothelin signaling pathway	7	2.290	0.080	0.772
PANTHER_PATHWAY	Notch signaling pathway	5	2.963	0.084	0.727
PANTHER_PATHWAY	Metabotropic glutamate receptor group II pathway	5	2.908	0.088	0.691
KEGG_PATHWAY	Neurotrophin signaling pathway	7	2.207	0.095	0.692
PANTHER_PATHWAY	Ionotropic glutamate receptor pathway	5	2.804	0.098	0.682
KEGG_PATHWAY	N-Glycan biosynthesis	4	3.564	0.100	0.679

## Discussion

In contrast to previous studies that focused mainly on the influence of maternal malnutrition on fetal neurodevelopment [1-3], [10], our study provides a striking view of how the epigenetic DNA methylation landscape of the thalamus and the hippocampus in postnatal individuals is modified in response to malnourishment. We describe a genome-wide, quantitative characterization of malnourishment-induced CpG methylation changes in postnatal mice. These data may serve as a useful resource for the neuroscience community and brain science studies.

Our study demonstrated several key aspects of malnourishment-induced epigenetic DNA modifications in mice. First, the global DNA methylation status in the thalamus and the hippocampus of postnatal malnourished mice and normal mice had significant differences. This told us that the mechanism of the malnourishment-induced epigenetic DNA modifications in the thalamus was different from the hippocampus. Second, our analysis revealed specific characteristics of the genomic DNA methylation distribution in the thalamus and the hippocampus of malnourished mice and normal mice. Third, our MSCC results provided direct profiling regarding the malnourishment-induced DNA methylation changes in both the thalamus and the hippocampus. Fourth, our study provided a large number of genes that were subjected to modulation by malnourishment at the level of DNA modification. The majority of these genes were associated with neuronal occurrence and development.

CpG-rich regions of DNA are known as CpG islands (CGIs), and most CGIs remain unmethylated [48]. Moreover, approximately 60% of mammalian genes have CGI promoters, and methylated CGIs play an important role in gene silencing during processing [49]. The best-known producer of epigenesis, DNA methylation, plays an important role in regulating gene expression to preserve local activity states [50]. Epigenesis is defined as heritable changes in gene expression that are not accompanied by changes in DNA sequence [51]. Thus, to reflect the DNA methylation landscape and distribution, we measured the methylation level in different regions of the genome. To gain more insight into whether the changes caused by methylation were present on the gene expression level, we performed comparisons between our MSCC data and the gene expression data (GDS1490). All our results were in accordance with the theoretical basic characteristics of DNA methylation modification in mammals, which confirmed the accuracy of our experiment.

It should be noted that when we used our criteria (∆MSCC > 25%) to screen out differential genes in the normal and famine groups, no genes were selected in the hippocampus, while 500 distinct genes were revealed in the thalamus. This may due to the selected CCGG methylated level in the hippocampus, which was much lower than that in the thalamus. Thus, a methylation level of >25% was difficult to achieve in the hippocampus. In this regard, further study of the hippocampus, which has vital roles in brain development, cognition, learning, and memory [52], would provide valuable information.

In the thalamus, the most significant identified relative pathway was long-term potentiation. It is a major form of long-lasting synaptic plasticity in the mammalian brain, which occurs by increasing synaptic strength, and is involved in information storage, and therefore, in learning and memory [53]. *Ppp1r1a*, *Ppp3r1*, *Raf1*, *Camk2b*, *Ppp3ca*, *Prkacb*, *Cacna1c*, *Plcb1*, and *Calm2* were the filtered genes in this pathway from our study. These genes are critically involved in neuronal formation and development. Among the 9 genes, *Camk2b* plays important roles in brain synaptic plasticity [54]. *Ppp3ca* is a tumor suppressor gene that functions in Alzheimer’s disease [25,26]. *Prkacb* is a protein kinase and is related to Alzheimer’s disease [16]. *Plcb1* is of critical importance in codifying neurotransmitter receptors and is associated with schizophrenia [46,47]. It is particularly noteworthy that the calcium channel, voltage-dependent, L-type, alpha 1C subunit (*Cacna1c*) gene contributes to many psychiatric disorders [55,56], specifically schizophrenia [40-42] and bipolar disorder [43-45].

Our study considered the global DNA methylation status of the thalamus and the hippocampus and provides a DNA methylation landscape of these two brain regions after they were modified by malnutrition. It also implicates DNA modification as an effective epigenetic regulator in postnatal brain maturation. Our data also indicate that malnutrition in postnatal individuals may increase the risk of developing psychiatric disorders such as Alzheimer’s disease, schizophrenia and bipolar disorder. Nonetheless, we believe that much more research on the functional verification of the related genes is necessary to obtain a better understanding of the pathogenesis of malnutrition.

## Conclusions

In this study, the thalamus and the hippocampus had different global DNA methylation statuses in postnatal malnourished mice and normal mice. Discriminable variations related to neuronal development and psychiatric disorders were also observed in the thalamus. Pathway analyses of the corresponding genes highlighted changes for 9 genes related to long-term potentiation (5.3-fold enrichment, P = 0.033). Our findings may help to differentiate the genome-wide DNA methylation status of different brain regions, and the results also indicate that postnatal malnutrition may increase the risk of psychiatric disorders.

## Abbreviations

5mC: 5-methylcytosine; NGS: Next-generation sequencing; MSCC: Methyl-sensitive cut counting; SLAC: Shanghai Laboratory Animal Co. Ltd; RFU: Relative fluorescence units; DMRs: Different methylated regions; ∆MSCC: Different levels of methylation changes; SD: Standard deviation; TSS: Transcription initiation site; LTP: Long-term potentiation; CGIs: CpG islands; Cacna1c: Calcium channel, voltage-dependent, L-type, alpha 1C subunit gene.

## Competing interests

The authors declare that they have no competing interests.

## Authors’ contributions

LH and YL supervised the experiment. DZZ and FTL designed the experimental protocol. XLW and FTL carried out the experiment. YL, XLW, DZZ, FTL, HZ, JYY, ZZ, DZ, YNW, LMT, LC, MYK, TW, GYF, XLQ, and JHS analyzed and discussed the experimental results. Finally, XLW, YL, and FTL wrote the manuscript. All authors read and approved the final manuscript.

## Supplementary Material

Additional file 1 Supplementary materials. Click here for file

Additional file 2: Table S1 MSCC results of the thalamus for all MSCC 30+ sites. Click here for file

Additional file 3: Table S2 MSCC results of the hippocampus for all MSCC 30+ sites. Click here for file

Additional file 4: Table S3 Selected 500 distinctive genes in the thalamus. Click here for file
