# Supplementary material for: DNA methylation profiling in the thalamus and hippocampus of postnatal malnourished mice, including effects related to long-term potentiation
Source: BMC Neurosci. 2014 Feb 20;15:31. doi: 10.1186/1471-2202-15-31 (PMC3941971; doi:10.1186/1471-2202-15-31)
Supplement: Additional file 1 — Supplementary materials. [file 1471-2202-15-31-S1.doc]

**Supplementary Information**

**Contents:**

Supplementary Methods

Supplementary Figures 1-3

**Supplementary Methods**

In MSCC library construction, for the *HpaII* Library, 2 ug of genomic DNA was digested with 20 units *HpaII* (New England Biolabs, Hitchin, UK) within 1×NEBuffer 1 in a 100 ul reaction. 1.66 ul of 10 uM adaptor A, 12 ul 10 mM ATP, and 120 units T4 DNA ligase (New England Biolabs, Hitchin, UK) was then added to the digested fragments. After digestion with 2 units *MmeI* (New England Biolabs, Hitchin, UK), 50 uM SAM, and 1×NEBuffer 4, the resulting fragments were ligated by 1.66 ul of 10 uM adaptor B, 6 ul 1 mM ATP, and 3 ul T4 DNA ligase. The mixture was then run on a 2% e-gel (Life Technologies, Carlsbad, CA) and the target band at ~140 bp was purified using QIAquick Gel Extraction Kit (QIAGEN, Hilden, Germany). PCR enrichment using primers matching the sequences of adaptor A and adaptor B was performed with conditions as follows: initial denaturation for 45 s at 98°C, followed by 8 cycles of (15 s at 98°C, 30 s at 60°C and 30 s at 72°C), followed by a final extension for 5 min at 72°C. The final PCR products were then purified using the QIAquick PCR Purification Kit (QIAGEN, Hilden, Germany). For the Inverse library, the fragment ends were deactivated by treating with Antarctic Phosphatase after digested with *HpaII*. Then *MspI* (New England Biolabs, Hitchin, UK) was used to digeste the preceding DNA, and followed by the same manner as the *HpaII* library.

1. b.


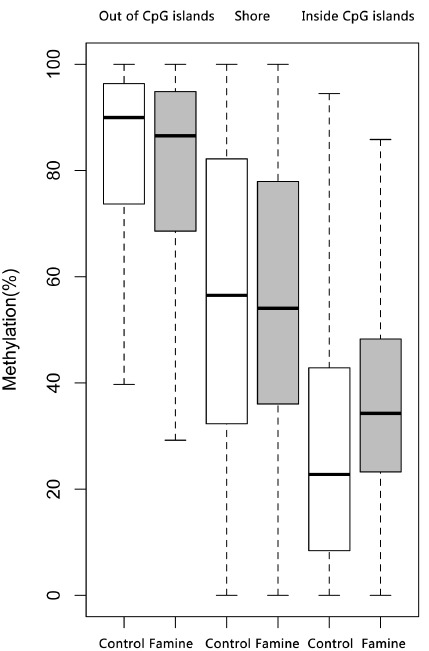

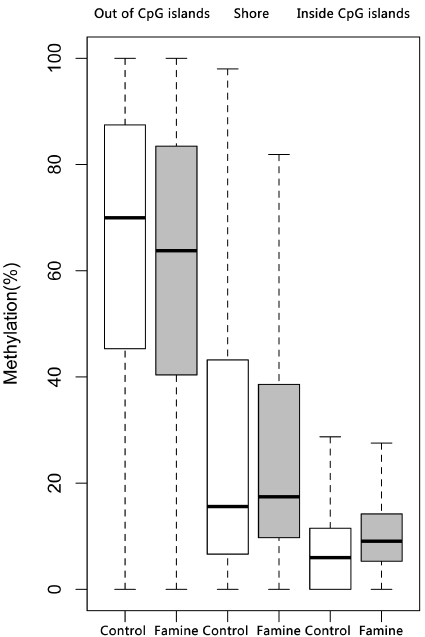


**Supplementary Figure 1.** The methylation level in three parts of CpG islands (control group in blank and famine group in gray) in the thalamus (a) and hippocampus (b).


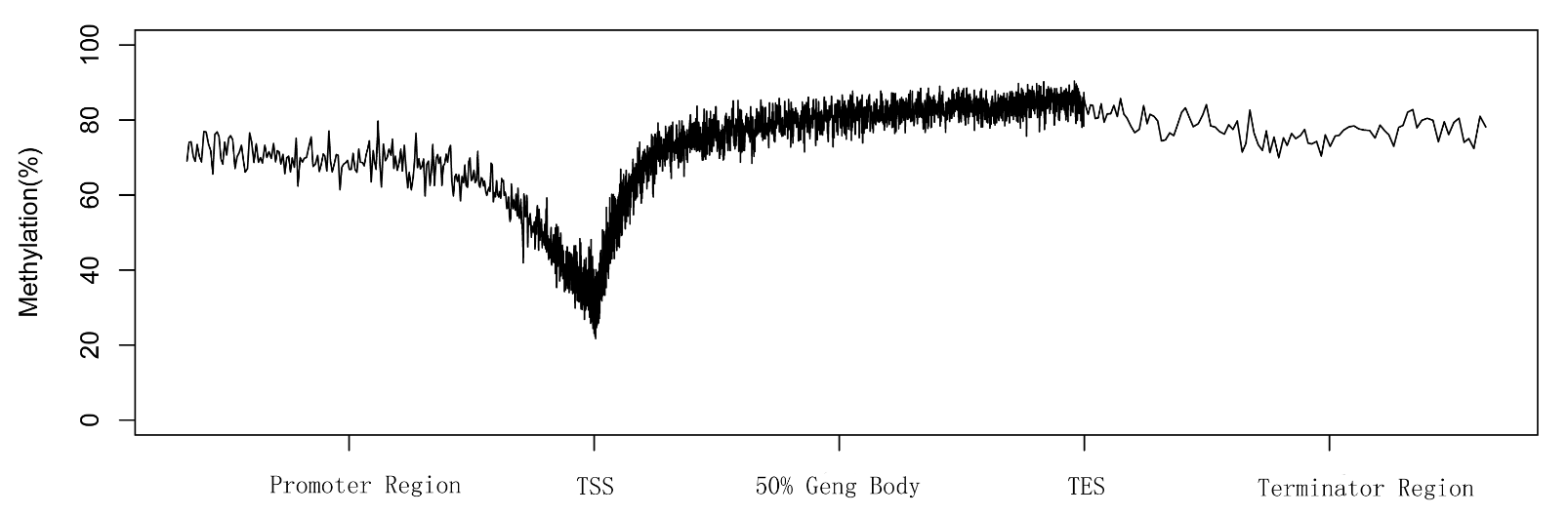


**Supplementary Figure 2.** Methylation level at the different positions of the whole genome of the thalamus. TSS: transcription initiation site; TES: transcription end site.

1. b.


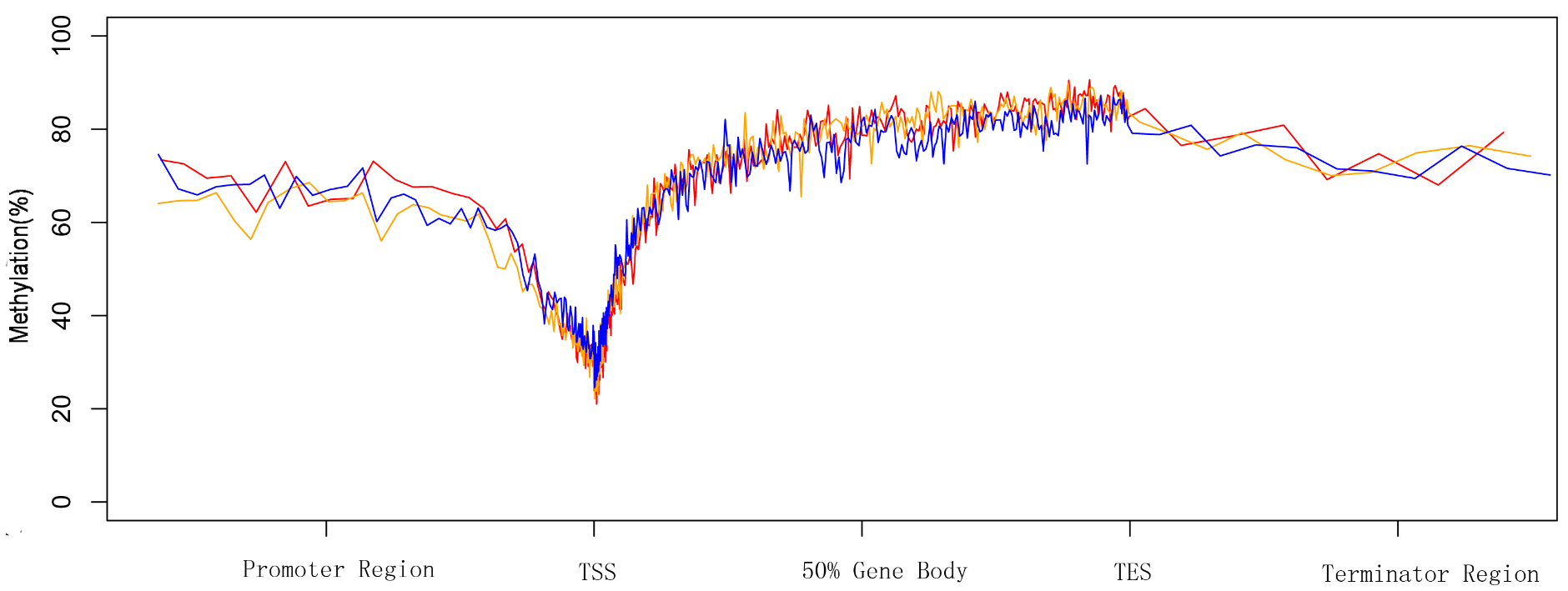

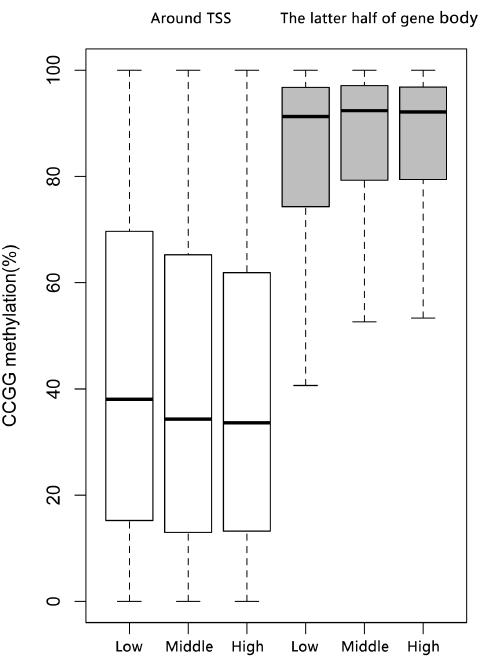


**Supplementary Figure 3.** NCBI gene expression data (GEO, GDS1490) combined with our MSCC methylation level of control group of the thalamus. (a) The red line showed high-expression genes, and the blue line stood for low-expression genes while the orange line meant moderate-expression genes. (b) CCGG methylation levels around TSS were showed in blank while the latter half of gene body were in gray.
